# Unveiling the Role of IGF-I in Fertility: Effect of Long-Acting Bovine Somatotropin (bST) on Terminal Follicular Development and Fertility during an Annual Reproductive Cycle in Sheep

**DOI:** 10.3390/ani14071097

**Published:** 2024-04-04

**Authors:** José Francisco Cox, Albert Carrasco, Felipe Navarrete, Rodrigo Allende, Fernando Saravia, Jesús Dorado

**Affiliations:** 1 Department of Animal Science, Faculty of Veterinary Sciences, Universidad de Concepción, Vicente Méndez 595, Chillán 3780000, Chilefsaravia@udec.cl (F.S.); 2 Department of Medicine and Animal Surgery, Faculty of Veterinary Medicine, University of Cordoba, Campus Rabanales, 14014 Cordoba, Spain

**Keywords:** bovine somatotropin, IGF-I, insulin, terminal follicular development, sheep

## Abstract

**Simple Summary:**

An understanding of the mechanisms involved in the nutritional regulation of the ovulation rate would increase sheep productivity and would improve animal feeding strategies and environmental footprints. The interaction of the metabolic axis controlling energy balance and the reproductive axis occurs at central and peripheric levels, ultimately signaling their influence directly in the reproductive tract. Growth Hormone (GH), insulin-like growth factors (IGF), and insulin are major peripheric players in this linkage. In addition to controlling energy homeostasis, their specific roles in modulating reproduction efficiency are unclear because they are functionally associated. Based on a single administration of long-acting bST, this study used a model that increased blood concentrations of IGF and kept basal that of insulin to assess, during an annual reproductive cycle, the effect of bST on terminal follicular development and oocyte competence for fertility. Results show that bST increased the number of lambs born from each ovulatory-sized follicle only during the breeding season, without affecting any other clinical marker. This model and the information gathered by this study allow a precise search for the molecular mechanisms involved in this effect.

**Abstract:**

The study aimed to assess the effect of long-acting bST treatment, in a dose that only increases IGF-I plasma concentrations, on ovarian and fertility markers of estrous synchronized ewes that were fed to keep their bodyweight. Three experiments were designed to evaluate this effect: in Experiment 1, 18 ewes were distributed in groups (bST 0, 30, 50 mg) to measure plasma IGF-I and insulin for 15 days; in Experiment 2, 92 ewes (5 replicates) in two groups (0 and 30 mg bST) were synchronized using a 6-day progesterone protocol during the breeding season to assess the effect of bST on follicular and luteal performances, estrous and ovulation, and fertility after mating. In Experiment 3, 50 ewes (3 replicates) were used to repeat the study before but during anestrus. Results indicate that 50 mg bST increased IGF-I and insulin plasma concentrations, but 30 mg bST only increased IGF-I concentrations; and that only during the breeding season did 30 mg bST increase the number of lambs born and the reproductive success of ovulatory-sized follicles compared to controls. This occurred without it affecting any other reproductive marker. In conclusion, 30 mg bST treatment may improve oocyte competence for fertility during the breeding season.

## 1. Introduction

Reproduction is a biological process that fundamentally relies on energy. Accordingly, the energy status affects every aspect of ruminant reproduction. Studies have shown that limiting energy can disrupt the reproductive process [1,2], while temporarily providing an excess of energy, a practice known as flushing, has been proven to increase the ovulation rate and lambing performance in sheep [3,4]. It appears evident that energy status is acutely monitored through endocrine and nutritional signals, which are evaluated both centrally and peripherally to modulate reproductive function [5,6,7]. While there is consensus on this general perspective, the precise signals and mechanisms that connect the metabolic and reproductive axes are yet to be fully understood. This understanding is crucial for improving the effectiveness of reproductive protocols and their impact on sheep productivity.

Gaining a deeper understanding of the mechanisms that drive the impact of flushing on lambing performance could improve reproductive protocols intended for its regulation. As such, terminal follicular development, defined as the FSH-dependent follicular stage of development [8,9], is a logical target to look for the peripheral influence of energy metabolism upon reproduction. This is because its course determines the ovulatory rate and the oocyte functional competence for fertilization, and development in ruminants [10,11,12]. Recent information has demonstrated that follicular dynamics is tightly regulated by the interplay between reproductive and metabolic factors; however, the specific signals that drive terminal follicular development and oocyte quality remain ambiguous [9,12,13].

The terminal follicular development can be programmed and monitored to pinpoint critical events that may be affected by metabolic influences, thereby enabling the identification of precise links between both axes. According to its driving influence, this follicular stage can be segmented into an FSH-dependent growth phase and an LH-dependent maturation phase [8,9]. The initial phase does not appear critical, given the limited central capacity to influence the FSH pituitary secretion and its consequences [14,15,16]. However, the LH-dependent phase of maturation seems more susceptible. This vulnerability is not only because most of the central influence of energy metabolism is mediated by the GnRH/LH pulse generator [2], but also because the follicle is exposed to a differentiation process that has several pathways that can compromise its functional competence [12,17,18]. In a recent study, Hattory et al. [8] recently referred to this as a tetralogy that includes greater capacity for estradiol production, activation of the IGF system, acquisition of LH dependence, and an antiapoptotic follicular microenvironment.

The role of the IGF system’s activation in the maturation phase of development makes it a potential link between the reproductive and metabolic axes. Indeed, the surge in free IGF is considered to be the initial event of follicular selection for dominance in cattle [19,20,21]. Furthermore, a decline in blood concentrations of IGF-I in ruminants under a negative energy balance is tied to a disruption in terminal follicular development and estradiol production [22,23,24].

Growth Hormone (GH), insulin-like growth factor I (IGF-I), and insulin are key regulatory signals in the regulation of energy homeostasis at the peripheral level. Consequently, their influence on follicular function in ruminants has been a matter of interest [25]. Although there is a gap in information regarding a direct role of GH on follicular regulation at this stage, more information is available for IGF (IGF-I and II) and insulin. Cumulative information suggests that, by acting on granulosa cells, they stimulate terminal follicular development and estradiol production. Moreover, they synergize with gonadotropins, enhancing the expression of FSH and LH receptors and modulating their subcellular pathways’ post-receptor activation [26,27]. 

IGF and insulin act on granulosa cells, probably through the IGF-I receptors (IGFIR), as recently shown in mice using knockout (KO) models [28]. The study found that mice deficient in IGFIR in granulosa cells were infertile, and their ovaries lacked late antral follicles. Additionally, mice with no insulin receptor expression showed no noticeable effect on fertility. Despite their structural similarities, IGF-I has about 6 times more affinity than IGF-II, and 100 times more affinity for its receptor than insulin [29]. Moreover, using KO-mice-specific models, it has also been shown that phenotypes lacking expression for papalisin-1 (PAPPA-A), also exhibit infertility problems [30].

The use of long-acting preparations of bovine somatotropin (bST) in ewes, maintaining a neutral energy balance, could potentially offer a valuable model for examining the activity of insulin-like growth factor (IGF) and insulin on terminal follicular development. bST can increase blood concentrations of both metabolic signals in ruminants [31]. This approach not only allows targeting specific stages of follicular development, but differing bST concentrations could also trigger varied responses in IGF-I and insulin secretion. This is because IGF-I secretion depends on the direct action of bST, while the insulin response to bST is a more intricate indirect effect (hyperglycemia and insulin resistance [32]). However, to date, neither approach has been tested. The influence of metabolic signals on reproductive function can be monitored clinically by morphological and functional markers. These markers are expressions of follicle and oocyte biological activities in response to regulatory signals, and their performances are usually compromised by the disruption of energy homeostasis [1,33,34].

Thus, this study aimed to evaluate the impact of bST administration on morphological, functional, and endocrine markers associated with terminal follicular development and reproductive success in sheep, over an annual reproductive cycle, based on ewes exhibiting synchronized follicular phases. We hypothesize that in ewes maintaining their body weight, adjusting the dosage of bST will elicit a differential response in IGF-I plasma concentrations compared to insulin, which will improve terminal follicular development and oocyte functional competence for fertility and development.

## 2. Materials and Methods

### 2.1. Animals and General Management

This study used an experimental flock of non-lactating and clinically sound Highlander ewes (*n* = 157) and Suffolk and Highlander rams (*n* = 6) 2–4 years old, identified by ear tags. It was conducted at the Faculty of Veterinary Sciences’ animal facilities, Universidad de Concepción, 36° south latitude, 71° west longitude. As a reference, the breeding season in Highlander in this area is between February and July [35]. This study followed the general procedures described by Cox et al. [31]. Animals were housed in a barn and kept in collective pens with an adequate space to rest and feed, with ventilation, dry bedding, and free access to fresh water. During experiments, sexually matured rams were kept in individual pens separated by fences that prevented direct contact; otherwise, they were kept in collective pens. During the day, animals were allowed access to a 4 ha paddock for grazing and exercise. Feeding at the research station was based on lucerne hay, oats grain, calf commercial concentrate, and mineral salts to maintain a BCS of around 3.0 (scale: 1–5; [36]). Moreover, animals were subjected to a preventive health program for endemic diseases. Housing and animal procedures followed standards approved by the Ethics Committee of the Faculty of Veterinary Sciences at the Universidad de Concepción (CBE-20-2022).

### 2.2. Estrous Detection 

Estrous detection during the experiments was conducted three times daily (08–09:00, 12:00–15:00, 18:00–20:00), by direct observation of tolerance of ewes to be mounted by rams. To facilitate sexual interaction, ewes were separated from the males, after a male rotation, ewes were reintroduced into the collective pen individually to register their sexual behavior, leaving behind ewes already identified as mated. The estrus presentation was registered as a middle point between the last negative period and the moment they exhibited ram receptivity. 

### 2.3. Blood Sampling and Endocrine Measures

Plasma concentrations of insulin, IGF-I, and progesterone were measured during the study. Blood samples (3 mL) were collected by jugular venipuncture into heparinized glass tubes and were maintained on ice (less than 2 h) until plasma collection. Plasma was obtained by centrifugation at 5 °C (1500× *g*, 20 min), and samples were labeled and stored at −20 °C until assayed. Insulin was measured by IRMA and IGF-I by a solid phase RIA, both using commercial kits validated for ruminants (DIASource, Louvain la Neuve, Belgium and CT Immunoassays SA, Louvain la Neuve, Belgium; respectively). The inter- and intra-assay CVs were <2.8% and <5.1%, respectively, and the limit of sensitivity was 3.4 ng/mL. Progesterone was measured by RIA, using also a commercial kit (PROG-RIA-CT, DiaSource, Louvain la Neuve, Belgium) validated for ruminants. The inter- and intra-assay CVs of this assay were 3.1% and 5.1%, respectively, and the limit of sensitivity was 0.05 ng/mL. 

### 2.4. Ovarian Ultrasound Measures

Follicles and corpora lutea (CLs) were evaluated by transrectal ovarian ultrasonography (US), using a 10 MHz linear array probe connected to a B-mode, real-time scanner (Honda 2010 Vet, Toyohashi, Japan). The transducer was fitted to a plastic rod that allowed the transrectal manipulation of the probe. Images were viewed at a magnification of ×2.0 with constant gain and focal point settings. The images of antral follicles and CLs were registered in videos, frozen as adequate, and measured in mm by internal calipers. At each examination, the relative position and dimension of follicles and CLs were sketched on ovarian charts. General ovarian features that were considered in this study included the number and the diameter of follicle ≥3.5 mm [37] at the end of progesterone treatment; the number and the diameter of ovulatory-size follicles (≥4.3 mm; [35]) 48 h later; and for ovulated ewes, the number and total area (mm) of CLs on day 7 (estrus = day 0).

### 2.5. General Definitions

The following definitions were used to characterize the ovarian and reproductive performance of experimental ewes: (a) estrous presentation (%): the percentage of ewes that exhibited estrus compared to those treated for estrous synchronization (ES); (b) ovulated ewes (%): the percentage of ewes that ovulated compared to those treated for ES; (c) ovulation efficiency (%): the percentage of follicles ≥4.3 mm on day 2 after CIDR that ovulate, based on CLs on day 7 after estrus; (d) total luteal area (mm): effective area of CLs ((diameter^2^/4) × π). In CLs that exhibited a cavity, the cavity area was subtracted from their area; (e) pregnancy rate (%): the percentage of ewes that were pregnant compared to those treated for ES; (f) lambing rate (%): the percentage of ewes that lambed compared to those that were pregnant; (g) fecundity rate (%): the percentage of lambs born compared to ewes treated for ES; (h) reproductive success (%): was used to establish the percentage of follicles ≥4.3 mm on day 2 that resulted in a lamb. 

### 2.6. Experiments

#### 2.6.1. Experiment 1: The Effect of bST Administration on Plasmatic Concentrations of IGF-I and Insulin in Sheep 

**Figure 1:**
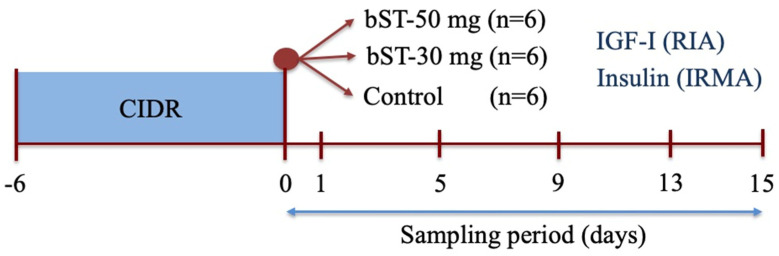
Protocol of blood collection (jugular puncture) for measuring IGF-I and insulin in plasma; IGF-I was measured by radioimmunoassay (RIA) and insulin by immunoradiometric assay (IRMA). The experimental group was synchronized by a short treatment of progesterone (CIDR).

To assess insulin and IGF-I profiles after bST, 18 ewes were synchronized during anestrus (December) by a treatment with a progesterone intravaginal insert (progesterone 0.3 g, CIDR G, InterAg, Hamilton, New Zealand) for 6 days. At the end of the treatment (day 0), each ewe was allocated into 3 groups: Group 1 was treated with 50 mg of bST each (s.c., Lactotropina^®^, Elanco, Eli Lilly, Ciudad de México, Mexico; bST-50, *n* = 6); Group 2 was treated with 30 mg bST (s.c., bST-30, *n* = 6); and Group 3 remained untreated (Control, *n* = 6). bST was placed in individual syringes and applied at the neck base. Blood samples were collected before morning feeding (9:00) on day 0 (before bST administration) and on days 1, 5, 9, 13, and 15 after bST treatment. Plasma was recovered as described and samples were labeled and stored until assayed for IGF-I (RIA) and insulin (IRMA) concentrations (Figure 1).

#### 2.6.2. Experiment 2: The Effect of bST Administration on Follicular Performance, Estrous Presentation, Ovulation Performance, Luteal Development, and Fertility after Mating in Sheep Treated for Estrous Synchronization during the Breeding Season 

To assess the influence of bST on terminal follicular development in synchronized follicular phases in sheep, between April and June, 92 ewes (5 replicates) were treated with progesterone (CIDR-G) for 6 days and prostaglandin F2α (0.15 mg DL-cloprostenol, Ciclase^®^, Syntex, Buenos Aires, Argentine) at CIDR removal. At CIDR insertion, the ewes, blocked by antral follicular counts (AFC), were allocated randomly into two groups that were kept mixed in collective pens (8–10 ewes each). One group was treated with 30 mg bST as above (Group BST, *n* = 46), and a second group remained untreated (Control, *n* = 46). At CIDR removal, a group of 4 rams of known fertility and libido was introduced alternately, keeping a single ram within the group (1:8–10 ram to ewe ratio), and they were kept rotating between pens at intervals of 6–14 h during a period of 4 days after CIDR. To assess follicular development, the number and diameter of follicles ≥3.5 mm and ≥4.3 mm in diameter were registered in each ewe at CIDR removal and 48 h later respectively. CL assessment included the number and total area of CL identified on day 7 after estrus. Mating activity was registered for estrous presentation and for pregnancy, and lastly, each ewe was mounted for at least two rams. To avoid unplanned pregnancies after mating for estrous detection, all ewes were treated with cloprostenol 5 days after the estrus. Pregnancy rate was carried out 32–35 days after estrus by transrectal US, and lambing performance was assessed to register the information on reproduction success rate. To assess the maintenance diet, ewes were weighed before morning feeding at CIDR insertion and on day 7 after the induced estrus, and their bodyweight were registered. 

**Figure 2:**
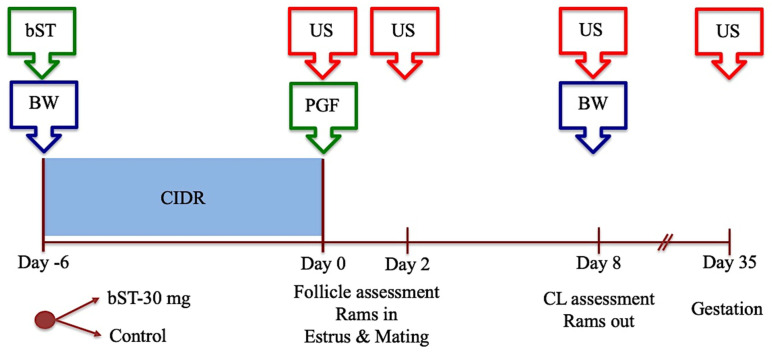
Experimental protocol for Experiments 2 and 3. Experimental groups were conformed at CIDR insertion. bST = bovine somatotropin; PGF = cloprostenol; BW = bodyweight assessment; US = transrectal ultrasound.

The effect of bST was evaluated according to the following parameters: the number and diameter of follicles ≥3.5 mm at CIDR removal and follicles ≥4.3 mm at 48 h later; interval to estrus (h from CIDR removal), estrous presentation (%); ovulated ewes, ovulation efficiency, CL development (number, luteal area (mm) and progesterone concentrations on day 7 after estrus), pregnancy, lambing and fecundity rates, and reproductive success. The experimental protocol is depicted in Figure 2.

#### 2.6.3. Experiment 3: The Effect of bST Administration on Follicular Performance, Estrous Presentation, Ovulation Performance, Luteal Development, and Fertility after Mating in Sheep Treated for Induction and Synchronization of Estrus during the Anestrous Season

The same setting used in Experiment 2 was utilized in this experiment. In brief, 50 ewes were synchronized between October and December by the 6-day progesterone protocol followed by the mating management described above and using the same rams. The group was blocked by AFC, and ewes were weighed at CIDR insertion, and randomly allocated into bST-treated (30 mg s.c., BST, *n* = 25) and untreated (Control, *n* = 25) groups. Both groups were housed in mixed groups in collective pens. At CIDR removal, rams were introduced into the groups, and they were managed as described above, during the same period. Follicular and CL development was assessed as before, and the same procedures were used for mating performance, pregnancy rate, lambing performance, and the control of bodyweight. Finally, the effect of bST was assessed using the same parameters utilized in Experiment 2.

### 2.7. Statistical Analysis

Values obtained were expressed as mean ± standard error of the mean (SEM). Data distributions were tested by the D’Agostino–Pearson test to assess their normality, and to proceed accordingly. IGF-I and insulin plasma sample concentrations among treatments were compared by a mixed-effect model (REML), and the cumulative secretion was compared by the Area Under the Curve (AUC) test. Normally distributed data were analyzed by non-paired t-test, whereas non-normally distributed data were compared by Mann–Whitney test. Binomial distributed data were compared by Chi-Square or Fisher Exact. A value of *p* < 0.05 was statistically significant. The Prism 10 (GraphPad Inc., La Jolla, CA, USA) statistical package was used for the foregoing analyses.

## 3. Results

### 3.1. Experiment 1. The Effect of bST on Plasma Concentrations of IGF-I and Insulin in Sheep

**Figure 3:**
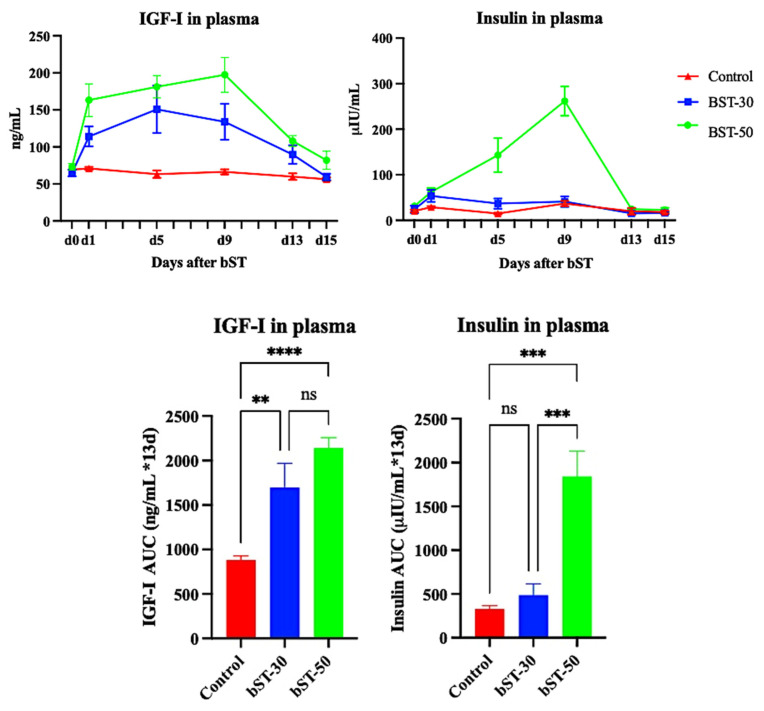
Daily and cumulative (AUC) concentrations of IGF-I and insulin in plasma after a single administration of 30 mg and 50 mg long-acting bST (Lactotropin^®^). An untreated group of ewes were left as control animals. ** *p* < 0.001; *** *p* < 0.0001; **** *p* < 0.00001; ns non-significant.

Plasma samples were obtained from 16 ewes allocated to experimental groups. One ewe exhibited a clinical condition and was removed from the experiment before ending the sampling (from the bST-30 group), and one exhibited outlier values for IGF-I (from the bST-50 group), so they were not considered for this experiment. A paired analysis was performed to assess whether values obtained in each sampling period were under the physiological range (using untreated ewes as a reference), and an AUC analysis was used to verify cumulative IGF-I and insulin secretion after bST. Results are shown in Figure 3.

The analysis of IGF-I concentrations showed that there was a significant effect of treatment (*p* < 0.0001), day (*p* < 0.0001), and the interaction between treatment and day (*p* < 0.0001). Values obtained in each sampling period showed no significant differences in plasma IGF-I between ewes from the Control vs. bST-30 groups in any period, but a tendency for plasma concentrations from day 1 to day 9 to differ was noted (*p* < 0.10); when Control ewes vs. BST-50 ewes were compared, IGF-I concentrations differed significantly from day 1 to day 13 (*p* < 0.05); and no differences in IGF-I concentrations were found between bST-treated ewes throughout the sampling period (*p* > 0.10). When cumulative IGF-I concentrations were compared between groups, IGF-I plasma concentrations were significantly different between ewes in the Control group vs. ewes from the BST-30 (*p* = 0.0025) and ewes from the bST-50 group (*p* < 0.0001); again, no significant differences were found between bST-treated groups (*p* = 0.106). 

The analysis of insulin concentrations showed that there was an effect of treatment *(p* < 0.001), day (*p* = 0.0003), and in the interaction between treatment and day (*p* < 0.0001). Values obtained for each sampling period showed that there were no significant differences in insulin concentrations in ewes from the Control and bST-30 groups in any sampling period; however, when the same was performed comparing ewes from both groups vs. ewes from the bST-50 group, the latter exhibited higher concentrations of insulin from day 9 (*p* = 0.01 and 0.007, respectively) to day 13 (*p* = 0.008 and 0.005, respectively). The AUC for cumulative concentrations of insulin until day 13 period indicates that there were no differences between ewes from the Control and bST-30 groups (*p* = 0.784), and both groups exhibited significantly lower concentrations of insulin compared to ewes from the bST-50 group (*p* = 0.0002 and *p* = 0.0007, respectively).

### 3.2. Experiment 2: The Effect of bST Administration on Follicular Performance, Estrous Presentation, Ovulation Performance, Luteal Development, and Fertility after Mating in Sheep Treated for Estrous Synchronization during the Breeding Season

The experiment utilized five replicates, with the last three also used to gather information on fertility performance and reproductive success. The global results are shown in Table 1. 

Collective results in Table 1 indicate that under bodyweight balance, the administration of bST increased the fecundity rate (*p* = 0.012) and the reproductive success (based on lambs born from ovulatory-sized follicles present at estrus; *p* < 0.001).

### 3.3. Experiment 3: The Effect of bST Administration on Follicular Performance, Estrous Presentation, Ovulation Performance, Luteal Development, and Fertility after Mating in Sheep Treated for Estrous Synchronization during the Anestrous Season

The same experiment as before was repeated during anestrous. The experiment had three replicates, and the results are shown in Table 2.

As in Experiment 2, ewes in both treatments maintained their bodyweight during the experiment (*p* > 0.10). Results in Table 2 indicate that ewes treated with bST exhibited similar performance with ewes left untreated based on the variety of morphological and functional markers considered in the study, including the reproductive success obtained (*p* > 0.10). 

## 4. Discussion

The main results of this study were that (a) the administration of bST in sheep influenced IGF-I and insulin plasma concentrations in a dosage-dependent manner. Either it elevated these concentrations for a duration of 13 days, or solely increased IGF-I plasma concentrations while preserving them within physiological ranges; and (b), the administration of bST to secure high but still physiological concentrations of IGF-I during terminal follicular development, increased the fecundity rate and the reproductive success of treated ewes only during the breeding season. It showed this without affecting any other clinical marker selected to monitor the ovarian and reproductive performance of Highlander ewes.

The administration of a zinc-base formulation of recombinant-bST to keep plasma concentration active for 14 days [38] directly increased plasma concentrations of IGF-I because of its ability to promote the IGF-I hepatic expression in ruminants [25,39,40]. In addition, bST also increased blood concentrations of insulin, as it promotes an increase in glucose concentrations and insulin resistance [32]. As expected, in this study ewes, treated with 50 mg bST exhibited similar profiles in IGF-I compared to earlier studies in sheep using the same bST [31]. However, it is new that by reducing the dose to 30 mg, ewes exhibited IGF-I plasma concentrations still within physiological concentrations when compared to untreated ewes in each sampling period, but the cumulative secretion throughout the period was significantly enhanced compared to untreated controls. However, insulin concentration remained unchanged in each sampling period and throughout the period compared to controls. 

This study also showed that combining a 30 mg dose of bST with a maintenance diet, aimed at stabilizing ewes’ bodyweight to minimize the confounding influence of metabolic signals on ovarian function and fertility [3,4], allowed for an experimental model that isolated the GH-related effect from insulin’s effect on terminal follicular development in sheep. This maintained IGF-I concentrations within physiological concentrations as both infra and supraphysiological IGF concentrations may disrupt cellular functions [41,42,43]. In addition, given that endocrine IGF-I concentrations primarily originate from GH activity on hepatocytes [35,40], IGF-I profiles can also be used to monitor bST’s biological activity after treatment.

Finally, we chose several clinical markers typically utilized to track the reproductive process, and two distinct periods of the annual reproductive season [44,45], to examine the impact of bST on the final stages of follicle development and fertility in sheep. We used the lambing performance as the primary endpoint of this study due to its well-recognized status as a marker of reproductive success. Based on this principle and adopting a similar strategy to Kleeman and Walker’s characterization of reproductive losses in Australian Merino flocks [46], we applied the concept of reproductive success based on ovulatory-sized follicles as a comprehensive marker of the functional competence of terminal follicular development.

The administration of bST (at a 30 mg dosage) during the breeding season increased the fecundity rate and reproductive success compared to untreated controls. It remains unclear how bST could have improved lamb production in ewes maintaining their bodyweight, and in the absence of significant differences in selected functional markers. Earlier reports have suggested a similar outcome under larger doses and alternative experimental approaches [47,48,49].

In addition, the increase in fecundity rate in the absence of differential effects in other selected markers used to monitor follicular function underscores the limitations of this method in assessing oocyte competence for fertility [12]. Studies in sheep have also shown that despite similar intervals to ovulation and CL development following estrous synchronization by similar protocols, ovulated follicles from the penultimate follicular wave early in the estrous cycle may exhibit diminished fertility potential [50]. Furthermore, in cattle, persistent follicles capable of ovulation and exhibiting normal post-ovulation CL development, still ovulate oocytes with lower fertility [51,52]. 

Although it is tempting to suggest that the stimulatory influence of bST was primarily exerted during the terminal phase of follicular development, this experimental design cannot discard the possibility of an effect of bST on embryo development [42,43]. However, the comparable interval to estrus, as an indicator of estradiol production, along with similar luteal area or progesterone plasma concentrations [53] displayed by both treated and control ewes, support the view that a key stimulatory influence occurred prior to ovulation. 

The intrafollicular microenvironment is clearly associated with oocyte competence for fertilization and development [10,12]. However, the mechanisms involved remain ambiguous, and according to these results, may include preserving the oocyte from aging [8,9,12]. Indeed, it has been suggested that a high fertility protocol for estrous synchronization in cows necessitates the triggering of follicular emergence during treatment to align oocyte functional competence with follicular competence [33]. 

On the other hand, when comparing the performance of Highlander breeds under commercial settings, the recorded lamb production aligns closely with that observed in bST-treated ewes in this study [35]. It is worth noting that in commercial flocks, conventional reproductive management is linked to a flushing period intended to enhance ovulation efficiency and lamb production [3,4]. It differs somewhat from our experimental setup, where the design aimed to maintain ewe energy balance, and there was less control over the origin of follicles involved in the ovulatory process [12,50,51].

The intrafollicular environment has been demonstrated to exert a significant, yet not fully understood, regulatory influence on an oocyte’s ability to acquire functional competence for fertility and development [9,10,12]. Despite the experimental evidence that shows that granulosa cells express mRNA for the somatotropin receptor and its protein in ruminants [54,55,56,57,58], and also that somatotropin promotes IGF-I and IGF-II secretion from thecal and granulosa cells, there is limited evidence that somatotropin directly regulates the maturation phase of terminal follicular development in ruminants (reviewed by [59] and [25]).

Thus, the prevailing view is that somatotropin acting on hepatic cells promotes the secretion of IGF-I and its binding complex (IGFBP-3 and ALS (acid labile subunit)), enabling its endocrine distribution. This rapidly equilibrates the follicular fluid concentration from where IGF-I exerts its effect on follicular cells and in the oocyte by binding to its receptor (IGF1R) [25,41,42,43,59,60]. The release of IGF-I from its binding complex, which neutralizes its activity, seems mediated by proteolytic degradation induced by PAPP-A2, a pappalysin protease expressed by granulosa cells from large antral follicles under FSH regulation [41,59,61]. Although the evidence supporting this view is consistent and associated with the clinical information gathered by ultrasound studies (reviewed by [19,44]), it is not possible to discard a key influence of a local activation of the IGF system (IGF-I and IGF-II). Both growth factors and IGFBP proteases are expressed in granulosa cells in differentiated antral follicles under the regulation of gonadotropins [8,13,27].

The increase in the fecundity rate and the reproductive success exhibited in this study by bST-treated ewes, despite no changes in follicular or functional marker performances, in the absence in follicular or functional marker performance, may well be the result of a direct effect on the oocyte, or an indirect effect through the follicular microenvironment supporting the oocyte competence, exhibited by IGF factors as suggested in other studies [9,10,12]. This speculation is based on the reported effects of IGF-I and II on granulosa cells as major pro-survival, steroidogenic, and differentiation regulatory factors [8,26,61,62].

The reproductive performance in experimental ewes during anestrus was depressed by the inhibitory photoperiod [45,63]. bST administration did not show significant effects either on any marker of ovarian function or in fertility. bST administration was expected to increase somatostatin release in the hypothalamus and, as has recently been shown, somatostatin is able to inhibit GnRH pulse frequency during anestrus in sheep, and also in other species [64,65]. The same authors [64] suggested that this neuropeptide could form a part of the steroid-independent inhibitory influence on GnRH pulse secretion, which adds to the inhibitory influence of estradiol during anestrus [63]. An altered LH pulse frequency would affect the paracrine regulation of follicular competence for terminal development and ovulation [10,11,66]. This observation highlights the hypothesis that LH pulse secretion plays a crucial regulatory role in integrating central and local factors that drive follicular maturation and oocyte competence for ovulation and fertility [12,31,67]. 

## 5. Conclusions

Cumulative information suggests that a single administration of bST at a low dose (30 mg) raises IGF-I plasma concentrations without modifying insulin concentration. As a result, treated ewes increased lamb production and reproductive success only during the reproductive season. Thus, the information gathered in the study partially supports the initial hypothesis that bST improves follicular and oocyte functional competence for development and fertility. 

## Figures and Tables

**Table 1:** The effect of bST administration on ovarian morphological and functional markers (Mean ± SEM), estrous presentation, ovulation rate, and reproductive success after mating in sheep treated with progesterone for estrous synchronization during the breeding season.

Parameters	bST	Control	*p* Value
Number of sheep (replicates)	46 (5)	46 (5)	
Body weight at day 0 (kg)	66.6 ± 2.24	69.2 ± 2.09	0.411
Body weight at day 15 (kg)	67.4 ± 2.25	69.5 ± 2.08	0.483
Follicles ≥ 3.5 at day 0:			
Number (*n*)	2.0 ± 0.18	2.2 ± 0.18	0.530
Diameter (mm)	4.7 ± 0.09	4.7 ± 0.09	0.738
Follicles ≥ 4.3 at day 2:			
Number (*n*)	2.1 ± 0.11	2.1 ± 0.10	0.771
Diameter (mm)	5.6 ± 0.08	5.6 ± 0.08	0.822
Estrous presentation (%)	100 (46/46)	97.8 (45/46)	>0.999
Interval CIDR-estrus (h)	37.0 ± 1.45	35.4 ± 1.61	0.451
Ovulated ewes (%)	100 (46/46)	100 (46/46)	1.000
Ovulation efficiency (%)	86.5 (45/52)	81.1 (43/53)	0.598
Corpora lutea at day 7:			
Number (*n*)	2.0 ± 0.10	1.9 ± 0.11	0.842
Total luteal area (mm)	171.5 ± 8.86	164.0 ± 9.54	0.567
Progesterone (ng/mL)	7.02 ± 0.76	6.93 ± 0.75	0.930
Pregnancy rate (%)	100 (23/23)	95.8 (23/24)	>0.999
Lambing rate (%)	100 (23/23)	91.3 (21/23)	0.709
Fecundity rate (%)	182.6 (42/23)	120.8 (29/24)	0.012
Reproductive success (%)	87.5 (42/48)	54.7 (29/53)	<0.001

**Table 2:** The effect of bST on ovarian morphological and functional markers (Mean ± SEM), estrous presentation, ovulation rate, and reproductive success after mating in sheep treated with progesterone for estrous synchronization during anestrous season.

Parameters	bST	Control	*p* Value
Number of sheep (replicates)	25 (3)	23 (3)	
Body weight at day 0 (kg)	60.9 ± 1.73	61.3 ± 2.16	0.876
Body weight at day 15 (kg)	62.0 ± 1.75	61.8 ± 2.23	0.947
Follicles ≥ 3.5 at day 0:			
Number (*n*)	2.2 ± 0.22	2.3 ± 0.19	0.734
Diameter (mm)	4.4 ± 0.08	4.4 ± 0.10	0.829
Follicles ≥ 4.3 at day 2:			
Number (*n*)	2.0 ± 0.15	2.3 ± 0.24	0.218
Diameter (mm)	5.1 ± 0.10	5.0 ± 0.11	0.847
Estrous presentation (%)	84.0 (21/25)	95.7 (22/23)	0.350
Interval CIDR-estrus (h)	40.4 ± 2.00	39.8 ± 2.5	0.803
Ovulated ewes (%)	84.0 (21/25)	95.7 (22/23)	0.350
Ovulation efficiency (%)	69.4 (34/49)	79.2 (42/53)	0.267
Corpora lutea at day 7:			
Number (*n*)	1.4 ± 0.16	1.8 ± 0.16	0.078
Total luteal area (mm)	130.7 ± 13.65	138.3 ± 10.08	0.458
Progesterone (ng/mL)	6.1 ± 0.54	6.1 ± 0.34	0.921
Pregnancy rate (%)	64.0 (16/25)	91.3 (21/23)	0.232
Lambing rate (%)	93.8 (15/16)	81.0 (17/21)	0.364
Fecundity rate (%)	84.0 (21/25)	104.3 (24/23)	0.414
Reproductive success (%)	42.9 (21/49)	45.3 (24/53)	0.844

## Data Availability

The datasets presented in this article are not readily available because they are part of an ongoing study. Requests to access the datasets should be directed to J.F.C.
